# Albumin for Adult ICU Patients With Shock: Protocol for the INCEPT‐Albumin Platform Trial Domain

**DOI:** 10.1111/aas.70203

**Published:** 2026-02-16

**Authors:** Tine Sylvest Meyhoff, Anders Perner, Praleene Sivapalan, Karen Louise Ellekjær, Aksel Karl Georg Jensen, Rikke Faebo Larsen, Maj‐Brit Nørregaard Kjær, Benjamin Skov Kaas‐Hansen, Theis Lange, Lasse Grønningsæter, Maria Cronhjort, Carmen Andrea Pfortmueller, Frederik Keus, Martin Ingi Sigurdsson, Erika Wilkman, Morten Hylander Møller, Anders Granholm

**Affiliations:** ^1^ Department of Intensive Care Copenhagen University Hospital – Rigshospitalet Copenhagen Denmark; ^2^ Collaboration for Research in Intensive Care (CRIC) Copenhagen Denmark; ^3^ Department of Clinical Medicine, Faculty of Health Sciences University of Copenhagen Copenhagen Denmark; ^4^ Section of Biostatistics, Department of Public Health University of Copenhagen Copenhagen Denmark; ^5^ Department of Anesthesiology, Division of Emergencies and Critical Care Oslo University Hospital Oslo Norway; ^6^ Department of Clinical Science and Education, Södersjukhuset Karolinska Institutet Stockholm Sweden; ^7^ Department of Clinical Sciences, Danderyd Hospital Karolinska Institutet Stockholm Sweden; ^8^ Department of Intensive Care Medicine, Inselspital University Hospital Bern Bern Switzerland; ^9^ Department of Critical Care, University Medical Center Groningen University of Groningen Groningen the Netherlands; ^10^ Faculty of Medicine University of Iceland Reykjavik Iceland; ^11^ Division of Anaesthesiology and Critical Care Medicine Landspitali – The National University Hospital of Reykjavik Reykjavik Iceland; ^12^ Perioperative and Intensive Care Helsinki University Hospital and University of Helsinki Helsinki Finland

**Keywords:** adaptive platform trial, albumin, fluid therapy, intensive care, randomised clinical trial, shock, trial protocol

## Abstract

**Background:**

Intravenous albumin is used for resuscitation of adult intensive care unit (ICU) patients with shock and substitution in those with hypoalbuminemia, which is common in critically ill patients. There is, however, substantial clinical practice variation as it is uncertain if albumin use improves patient‐important outcomes.

**Methods:**

The *INCEPT‐Albumin* domain is an investigator‐initiated, open‐label domain with an integrated feasibility phase on the international, pragmatic, parallel‐group, randomised, embedded, multifactorial, adaptive *Intensive Care Platform Trial (INCEPT)*. Adult acutely admitted ICU patients with shock irrespective of its cause will be randomised to albumin versus no albumin use for resuscitation and substitution while in the ICU for a maximum of 90 days. The primary outcome is days alive without life support at 30 days. Secondary outcomes include 30‐, 90‐, and 180‐day all‐cause mortality; days alive without life support at 90 days; days alive out of hospital at 30 and 90 days; days free of delirium at 30 days; health‐related quality of life and cognitive function at 180 days; and serious adverse reactions at 30 and 90 days. Analyses will primarily be conducted in the intention‐to‐treat population using Bayesian statistical models with neutral, weakly informative priors. We will assess feasibility after 200 participants and conduct adaptive analyses after follow‐up for the primary outcome of 1000 participants and every additional 250 participants to a maximum of 10,000, with adaptive stopping for superiority/inferiority and practical equivalence (mean difference in the primary outcome < 1 day). Allocation will initially be equal, followed by response‐adaptive randomisation with minimum 40% allocation to each arm. Expected sample sizes across different scenarios range from 1137 to 3547 participants, with approximately 100% probabilities of conclusiveness across scenarios.

**Conclusions:**

*INCEPT‐Albumin* will with high probability provide conclusive results and inform clinical practice regarding albumin versus no albumin use in adult acutely admitted ICU patients with shock.

## 1 Introduction

Shock is life‐threatening circulatory failure resulting in inadequate tissue perfusion most frequently caused by sepsis, severe bleeding, or heart failure [1]. One in three patients in the intensive care unit (ICU) have shock [2], and many will not survive to hospital discharge [3, 4]. Management strategies often involve fluid resuscitation, circulatory support (i.e., vasopressors or inotropes), and treatment of the underlying cause [1]. Intravenous albumin solutions are derived from human plasma [5] and are often given during fluid resuscitation of patients with shock [6]. By being a colloid solution, albumin may have volume‐sparing effects compared to crystalloid solutions [5]. Albumin may also be used to correct hypoalbuminemia, which is associated with poor outcomes [7].

A systematic review with meta‐analysis of 20 randomised clinical trials (RCTs) including 13,047 critically ill patients found little to no differences in 30‐ or 90‐day mortality when comparing colloids (albumin or fresh‐frozen plasma) to crystalloids (moderate certainty of evidence) [8]. Another systematic review concluded that albumin may have beneficial effects on fluid balance and haemodynamic endpoints compared to crystalloids in ICU patients [9]. Results regarding mortality were uncertain, although mostly compatible with small benefits with albumin use [9]. The results from both meta‐analyses are primarily driven by two large RCTs, both suggesting heterogeneous intervention effects with albumin [10, 11]. In the *Saline* versus *Albumin Fluid Evaluation (SAFE)* trial [11], ICU patients with traumatic brain injury randomised to albumin had higher 28‐day mortality than those randomised to saline (relative risk [RR] 1.62, 95% confidence interval [CI]: 1.12–2.34), while lower 28‐day mortality was suggested in those with severe sepsis randomised to albumin (RR 0.87, 95% CI: 0.74 to 1.02) [11]. In the *Albumin Italian Sepsis (ALBIOS)* trial [10], ICU patients were randomised to crystalloids and 20% albumin (for resuscitation and to maintain daily albumin levels at ≥ 30 g/L) versus crystalloids alone, with little to no overall differences in outcomes [10]. A *post hoc* subgroup analysis of 90‐day mortality (a secondary outcome) indicated benefit of albumin in those with septic shock as compared to those with sepsis without shock (RR for septic shock 0.87, 95% CI: 0.77 to 0.99; RR for sepsis without shock 1.13, 95% CI: 0.92 to 1.39; *p* value for heterogeneity 0.03) [10].

Due to uncertain evidence, there are substantial discrepancies and vagueness in recent clinical practice guidelines. The 2021 *Surviving Sepsis Campaign* guideline suggests using albumin in patients with sepsis or septic shock who have received large volumes of crystalloids (conditional recommendation, moderate certainty evidence) [12]. The *International Collaboration for Transfusion Medicine* guideline suggests not using albumin for first‐line resuscitation or to increase serum albumin levels in critically ill patients (conditional recommendation, moderate certainty evidence) [13]. The 2024 *European Society of Intensive Care Medicine* guideline suggests using crystalloids rather than albumin for volume expansion in critically ill patients in general (conditional recommendation, moderate certainty evidence) [14].

There is substantial practice variation for the use of albumin in shock. In the *Conservative* versus *Liberal Fluid Therapy in Septic Shock (CLASSIC)* trial, 45% of participants received albumin [3], with varying practices between sites beyond what could be explained by patient characteristics [15]. In a recent survey among 1248 ICU physicians across 21 countries, 26% reported almost always or frequently using albumin in patients with shock, while 40% reported rarely or never using it [6]. More than 90% of respondents supported enrolling ICU patients with shock in an RCT comparing albumin versus no albumin for resuscitation and substitution [6].

In summary, albumin given to adults with shock may have desirable effects on circulatory parameters and fluid balance, but it is uncertain if these effects translate into improved patient‐important outcomes. There is both clinical equipoise and support amongst ICU physicians for an RCT assessing albumin use in ICU patients with shock.

The *INCEPT‐Albumin* domain will assess if albumin use for resuscitation and substitution increases the number of days alive without life support at 30 days and improves other patient‐important outcomes compared with no albumin use in adult acutely admitted ICU patients with shock. We hypothesise that the use of albumin may result in more days alive without life support at 30 days without harmful adverse effects.

## 2 Methods

### 2.1 Design, Approvals, and Consent

The *INCEPT‐Albumin* domain is an investigator‐initiated, open‐label domain [16] with an integrated feasibility phase on the pragmatic, parallel‐group, randomised, embedded, multifactorial, international, adaptive *Intensive Care Platform Trial (INCEPT)* [17]. The full core protocol and domain‐specific appendices for all approved domains are available at the trial website (www.incept.dk) [18]; these documents include additional details, variable definitions, and completed reporting checklists (*Standard Protocol Items: Recommendations for Interventional Trials [SPIRIT]* [19] and *Consolidated Standards Of Reporting Trials, Adaptive designs Extension [CONSORT‐ACE]* [20]).

*INCEPT* and *INCEPT‐Albumin* have been approved by the Danish competent authorities and publicly registered before initiation (approvals and identifiers: EUCT number: 2024‐516208‐41‐00; ClinicalTrials.gov identifier: NCT06667999; Universal Trial Number: U1111‐1313‐8171); additional approvals will be obtained as required before initiation in other countries. Screening for *INCEPT‐Albumin* started on 26 June 2025.

*INCEPT* is an emergency medical trial generally enrolling participants without prior consent, followed by informed consent to continue in each domain from legal surrogates and participants as soon as possible; consent may be withdrawn at any time without explanation on a domain‐basis [17, 18].

### 2.2 Eligibility Criteria

Adult (≥ 18 years) acutely admitted ICU patients will be screened for *INCEPT‐Albumin* if both interventions are considered clinically appropriate and they have shock (ongoing infusion of a vasopressor/inotrope *and* plasma lactate ≥ 2 mmol/L within the last 3 h), irrespective of cause. Patients will be excluded for the following reasons: legal consent expected to be unobtainable; under coercive measures; receipt of albumin during the current ICU stay; traumatic brain injury; known pregnancy; known religious objection to albumin; known albumin allergy; or inclusion in another interventional trial or *INCEPT* domain where co‐enrolment with *INCEPT‐Albumin* is not permitted. Patients may only be randomised once to *INCEPT‐Albumin*.

### 2.3 Interventions

The intervention period is from randomisation to a maximum of 90 days while in the ICU, including transfers or readmissions to participating ICUs during this period.

#### 2.3.1 Albumin Use in the ICU (Intervention)

Albumin should be used during circulatory failure in addition to crystalloids (resuscitation) and for substitution in case of suspected or overt albumin loss, or plasma albumin levels ≤ 25 g/L. The concentration, dose, and timing are at the treating clinicians' discretion.

#### 2.3.2 No Albumin Use in the ICU (Control)

Albumin should not be used but may be considered in the following special circumstances: large ascites drainage (> 1 L tapped), spontaneous bacterial peritonitis, and hepatorenal syndrome.

#### 2.3.3 Co‐Interventions

Co‐interventions will be at the discretion of the treating clinicians (except for randomised comparisons in other *INCEPT* domains or trials with approved co‐enrolment [17, 18]).

### 2.4 Outcomes

The primary outcome also guiding all adaptations is days alive without life support (invasive mechanical ventilation, continuous use of vasopressors/inotropes, or renal replacement therapy) at 30 days, with non‐survivors assigned 0 days [21]. Secondary outcomes include the remaining *INCEPT* core outcomes and domain‐specific safety outcomes (severe anaphylactic reactions and major bleeding) [17, 18] (Table 1).

**TABLE 1:** Outcome data.

Outcome	Outcome data by intervention^a^ descriptive data [n/N (%) or median (IQR)] *model estimate [probability or mean (95% CrI)]*		Intervention effect estimates^b^		Probabilities of each intervention being best/better^c^	
	Albumin use (*N* = #)	No albumin use (*N* = #)	Absolute difference (MD/RD, 95% CrI)	Relative difference (RoM/RR, 95% CrI)	Albumin use	No albumin use
Days alive without life support at day 30^d^ (primary and guiding outcome)	#.# (#.# to #.#) *#.# (#.# to #.#)*	#.# (#.# to #.#) *#.# (#.# to #.#)*	#.# (#.# to #.#)	#.## (#.## to #.##)	#.##%	#.##%
Days alive out of hospital at day 30^d^	#.# (#.# to #.#) *#.# (#.# to #.#)*	#.# (#.# to #.#) *#.# (#.# to #.#)*	#.# (#.# to #.#)	#.## (#.## to #.##)	#.##%	#.##%
Days free of delirium at day 30^d^	#.# (#.# to #.#) *#.# (#.# to #.#)*	#.# (#.# to #.#) *#.# (#.# to #.#)*	#.# (#.# to #.#)	#.## (#.## to #.##)	#.##%	#.##%
All‐cause 30‐day mortality	n/N (#.#%) *#.#% (#.# to #.#)*	n/N (#.#) *#.#% (#.# to #.#)*	#.#%‐points (#.# to #.#)	#.## (#.## to #.##)	#.##%	#.##%
All‐cause 90‐day mortality	n/N (#.#%) *#.#% (#.# to #.#)*	n/N (#.#%) *#.#% (#.# to #.#)*	#.#%‐points (#.# to #.#)	#.## (#.## to #.##)	#.##%	#.##%
Days alive without life support at day 90^d^	#.# (#.# to #.#) *#.# (#.# to #.#)*	#.# (#.# to #.#) *#.# (#.# to #.#)*	#.# (#.# to #.#)	#.## (#.## to #.##)	#.##%	#.##%
Days alive out of hospital at day 90^d^	#.# (#.# to #.#) *#.# (#.# to #.#)*	#.# (#.# to #.#) *#.# (#.# to #.#)*	#.# (#.# to #.#)	#.## (#.## to #.##)	#.##%	#.##%
All‐cause 180‐day mortality	n/N (#.#%) *#.#% (#.# to #.#)*	n/N (#.#%) *#.#% (#.# to #.#)*	#.#%‐points (#.# to #.#)	#.## (#.## to #.##)	#.##%	#.##%
EQ‐5D‐5L index values [22] at day 180^e^	#.## (#.## to #.##) *#.## (#.## to #.##)*	#.## (#.## to #.##) *#.## (#.## to #.##)*	#.## (#.## to #.##)	#.## (#.## to #.##)	#.##%	#.##%
EQ VAS [22] at day 180^e^	#.# (#.# to #.#) *#.# (#.# to #.#)*	#.# (#.# to #.#) *#.# (#.# to #.#)*	#.# (#.# to #.#)	#.## (#.## to #.##)	#.##%	#.##%
Cognitive function (Mini MoCA) at day 180^f^	#.# (#.# to #.#) *#.# (#.# to #.#)*	#.# (#.# to #.#) *#.# (#.# to #.#)*	#.# (#.# to #.#)	#.## (#.## to #.##)	#.##%	#.##%
One or more domain‐specific safety outcomes at day 30^g^	n/N (#.#%) *#.#% (#.# to #.#)*	n/N (#.#%) *#.#% (#.# to #.#)*	#.#%‐points (#.# to #.#)	#.## (#.## to #.##)	#.##%	#.##%
One or more domain‐specific safety outcomes at day 90^g^	n/N (#.#%) *#.#% (#.# to #.#)*	n/N (#.#%) *#.#% (#.# to #.#)*	#.#%‐points (#.# to #.#)	#.## (#.## to #.##)	#.##%	#.##%

*Note:* Mock table illustrating how we plan to present data from the final, primary analyses of all clinical outcomes in the *INCEPT‐Albumin* domain. All model‐based estimates, intervention effects, and probabilities are based on the sample‐average posterior distributions for the outcome of interest. Definitions of all outcomes are provided in the core protocol [17] and full domain‐specific appendix [18].

Abbreviations: CrI: credible interval; MD: mean difference; n and N: n denotes number of participants with the outcome, N denotes the total number of participants in each arm; RD: (absolute) risk difference; RoM: ratio of means; RR: relative risk.

^a^ For both interventions, descriptive outcome data (counts with percentages for binary outcomes, medians with interquartile ranges for continuous/count outcomes) and estimated sample‐average probabilities (binary outcomes) or mean values (continuous/count outcomes) from the primary models will be presented with 95% CrIs. The counts and proportions of participants with missing data for each separate outcome variable will be reported, with descriptive data and model estimates based on multiply imputed data unless complete.

^b^ Sample‐average intervention effect estimates will be presented as between‐arm comparisons with the no albumin use (control) arm considered the reference.

^c^ The probabilities of one intervention being superior and the other being inferior will be presented, with the reference arm chosen as outlined in *b*. Probabilities of effect sizes smaller than the threshold(s) for practical equivalence will also be presented along with the probabilities of intervention effects larger than the practical equivalence threshold(s) in both directions. For all outcomes, the complete posterior distributions of the primary set of sample‐average intervention effects and the corresponding probabilities of *all* effect sizes will be visualised.

^d^ All *days alive without X*‐type outcomes will be reported with non‐survivors assigned 0 days primarily, and using actual values without penalisation of death in sensitivity analyses [21].

^e^ EQ‐5D‐5L index values will primarily be calculated using national value sets. Non‐survivors will be assigned 0 for EQ‐5D‐5L index values (corresponding to a health quality as bad as being dead) [22] and EQ VAS (corresponding to the worst possible value) [22], with secondary analyses conducted in survivors only.

^f^ Cognitive function assessed using the *Montreal Cognitive Assessment test 5‐minute version, v2.1 (“Mini MoCA”)* [23], with non‐survivors assigned 0 (worst possible value) in the primary analyses and in survivors only in secondary analyses.

^g^ One or more domain‐specific safety outcomes include severe anaphylactic reactions registered in participating ICUs and major bleedings registered in‐hospital.

### 2.5 Randomisation and Allocation Concealment

Allocation will initially be equal and stratified for site and shock type (septic shock versus other types of shock), followed by simple (unstratified) restricted response‐adaptive randomisation [24] after the first adaptive analysis based on each arm's probability of being superior and with minimum 40% allocation to each arm [17]. Allocation in *INCEPT‐Albumin* is independent of other domains, and allocation concealment is secured via an online electronic system.

### 2.6 Protocol Adherence, Feasibility, and Separation

Protocol violations (i.e., no albumin given during resuscitation or as substitution when the substitution criteria are present in the albumin use arm, or albumin given without one of the special circumstances being present in the no albumin use arm) will be registered and centrally monitored. Treating clinicians may deviate from the protocol at any time to ensure participant safety if continued protocol adherence is assessed to lead to suboptimal care.

An integrated feasibility phase including the first 200 participants will assess feasibility outcomes including separation (Table 2). The domain will continue without alterations if all feasibility criteria (Table 2) are met; otherwise, the domain management committee will decide if modifications of the domain are required.

**TABLE 2:** Feasibility outcomes.

Name	Definition/operationalisation	Feasibility criteria
Separation of mean volumes	Difference in mean albumin^a^ volumes between arms	≥ 500 mL^b^
Separation of median volumes	Difference in median albumin^a^ volumes between arms	≥ 500 mL^b^
Protocol adherence in albumin arm	Proportion of participants receiving ≥ 100 mL albumin^a^ in the albumin arm	> 90%
Protocol adherence in the no albumin arm	Proportion of participants receiving ≥ 100 ml albumin^a^ in the no albumin arm	< 10%
Feasibility phase completion	Time to completion of enrolment for the feasibility phase	< 9 months
Recruitment proportion	Percentage of patients screened for *INCEPT‐Albumin* who are included in the domain	> 50%
Participants without consent	Percentage of participants for whom any consenting party do not consent to continued collection of data	< 5%
Protocol violations	Combined percentage of patients who did not receive albumin in the albumin arm, and those who did in the no albumin arm	< 17%^c^
Retention proportion	Percentage of participants with primary outcome data within maximum 15 days of follow‐up	≥ 95%

*Note:* Overview of feasibility outcomes, including definitions and feasibility criteria.

^a^ Total volumes (regardless of reasons for administering, including albumin administered in the no albumin arm under the special circumstances where this is permitted) all concentrations combined.

^b^ Half of the difference observed in the *Albumin Italian Sepsis (ALBIOS)* trial [10].

^c^ Proportion participants with protocol violations in the *Conservative* versus *Liberal Fluid Therapy in Septic Shock (CLASSIC)* trial [3].

### 2.7 Statistical Methods

*INCEPT‐Albumin* will use Bayesian statistical methods [25] with neutral, weakly informative priors, with adaptive stopping and response‐adaptive randomisation and probabilistic interpretation of results [17].

#### 2.7.1 Analysis Sets and Primary Estimand

Analyses will primarily be conducted in the intention‐to‐treat population [17], with secondary analyses conducted in the per‐protocol population (i.e., excluding participants with protocol violations). The primary estimand in *INCEPT‐Albumin* is the sample‐average mean difference in days alive without life support at 30 days with albumin versus no albumin in acutely admitted adult ICU patients with shock, according to treatment allocation regardless of protocol adherence and with non‐survivors assigned 0 days.

#### 2.7.2 Adaptive Analyses and Adaptations

Adaptive analyses will be conducted on the first workday monthly after completion of follow‐up and data collection/validation for the primary outcome for the first 1000 participants and every 250 additional participants until a maximum sample size of 10,000 participants. Constant, symmetrical stopping rules for superiority/inferiority of 99.54% and 0.46% are used; these stopping rules have been calibrated using simulations to ensure a type 1 error rate for the primary outcome of ~5% [24, 26]. The domain will be stopped for practical equivalence if there is > 90% posterior probability that the mean difference in the primary outcome is < 1 day [24, 26]. Stopping rules are binding [27]. Response‐adaptive randomisation based on each arm's probability of being superior will be used after the first adaptive analysis, with minimum allocation of 40% to each arm [17, 24, 26].

#### 2.7.3 Descriptive Statistics

Baseline data (Table 3) separation data, and outcome data (Table 1) will be presented for each arm using medians with interquartile ranges for numeric data and counts with percentages for categorical data.

**TABLE 3:** Baseline data.

Characteristic	Albumin use (*N* = #)	No albumin use (*N* = #)
Country of enrolment		
Denmark	*n* (#.#%)	*n* (#.#%)
*[Each participating country listed separately in the domain report(s)]*	*n* (#.#%)	*n* (#.#%)
Age, median (IQR), years	## (## to ##)	## (## to ##)
Sex		
Female	*n* (#.#%)	*n* (#.#%)
Male	*n* (#.#%)	*n* (#.#%)
Weight, median (IQR), kg	## (## to ##)	## (## to ##)
Height, median (IQR), m	#.## (#.## to #.##)	#.## (#.## to #.##)
Use of invasive mechanical ventilation	*n* (#.#%)	*n* (#.#%)
Use of vasopressors/inotropes	*n* (#.#%)	*n* (#.#%)
Use of renal replacement therapy	*n* (#.#%)	*n* (#.#%)
Limitations of care	*n* (#.#%)	*n* (#.#%)
Co‐existing conditions		
Active haematological malignancy or metastatic cancer	*n* (#.#%)	*n* (#.#%)
History of ischaemic heart disease or heart failure	*n* (#.#%)	*n* (#.#%)
Diabetes mellitus	*n* (#.#%)	*n* (#.#%)
Chronic pulmonary disease	*n* (#.#%)	*n* (#.#%)
Chronic liver disease	*n* (#.#%)	*n* (#.#%)
Known use of immunosuppressive therapy within the last 3 months	*n* (#.#%)	*n* (#.#%)
Previous organ transplantation	*n* (#.#%)	*n* (#.#%)
Chronic dialysis	*n* (#.#%)	*n* (#.#%)
Treatment with antipsychotics at hospital admission	*n* (#.#%)	*n* (#.#%)
Primary cause of shock		
Septic	*n* (#.#%)	*n* (#.#%)
Cardiogenic	*n* (#.#%)	*n* (#.#%)
Haemorrhagic or traumatic	*n* (#.#%)	*n* (#.#%)
Other (e.g, neurogenic, anaphylactic, burn and obstructive shock)	*n* (#.#%)	*n* (#.#%)
Acute surgery within 7 days prior to randomisation	*n (#.#%)*	*n (#.#%)*
SMS‐ICU,^a^ median (IQR)	## (## to ##)	## (## to ##)
Predicted 90‐day mortality,^a^ median (IQR), %	## (## to ##)	## (## to ##)
Clinical Frailty Scale,^b^ median (IQR)	## (## to ##)	## (## to ##)
Lowest systolic blood pressure in the 24 h preceding randomisation, median (IQR), mmHg	## (## to ##)	## (## to ##)
Highest plasma lactate in the 24 h prior to randomisation, median (IQR), mmol/L	## (## to ##)	## (## to ##)
Highest plasma creatinine in the 24 h prior to randomisation, median (IQR), μmol/L	## (## to ##)	## (## to ##)
Lowest plasma albumin within the last 24 h, median (IQR), g/L	## (## to ##)	## (## to ##)
IV fluid volumes administered in the 24 h before randomisation, median (IQR), mL	## (## to ##)	## (## to ##)

*Note:* Mock table illustrating how we plan to present baseline data. Binary or categorical variables are presented as numbers with percentages; numerical variables are presented as medians with interquartile ranges. Definitions of all baseline variables are provided in the core protocol [17] and full domain‐specific appendix [18].

Abbreviations: IQR: interquartile range, mmHg: millimetres of mercury, mmol/L: millimoles per litre, N or n: total counts or counts, SMS‐ICU: *Simplified Mortality Score for the Intensive Care Unit* [28], μmol/L: micromoles per litre.

^a^ SMS‐ICU [28] is a severity score (range 0–42 points) and prediction model, with higher values indicating more severe illness and higher risks of 90‐day mortality.

^b^ Assessed by investigators using the Clinical Frailty Scale v2.0 [29, 30] ranging from 1 (very fit) to 9 (terminally ill).

#### 2.7.4 Primary Analyses

All outcomes will be analysed using Bayesian linear and logistic regression models adjusted for the stratification variables (site and shock type) and the following additional baseline variables: age (modelled using a linear and a quadratic term), sex, haematologic malignancy or metastatic cancer, acute surgery within 7 days prior to randomisation, invasive mechanical ventilation, renal replacement therapy within 72 h prior to randomisation, and time period (with a new period defined after each adaptation) [17]. Analyses will primarily use neutral, weakly informative priors not favouring any intervention, with sensitivity analyses using neutral, more informative (sceptical) priors and neutral, less informative priors, as well as evidence‐based priors for outcomes with relevant external evidence (exact priors presented in the full domain‐specific appendix [18]). Results will be presented as sample‐average estimates and intervention effects on the absolute (mean difference or risk difference) and relative (ratio of means or relative risk) scales, with the absolute summary measures being the primary [17], all calculated using G‐computation with the posterior probability distributions [17, 31]. Point estimates, 95% credible intervals, and probabilities of any benefit/harm (i.e., superiority/inferiority of either intervention, regardless of the size of the difference), and—for the primary outcome—probabilities of practical equivalence and differences larger than the equivalence threshold in both directions will be calculated, as outlined in the core protocol [17]. Model fitting and model diagnostics are described in the full core protocol [17, 18].

#### 2.7.5 Heterogeneous Intervention Effects and Interactions

Once the domain is stopped, potential heterogeneous intervention effects [32] for the primary outcome according to the baseline characteristics in Table 4 will be assessed on both the absolute and relative scales. For all analyses of heterogeneous intervention effects, results will be visualised with 95% credible intervals and interpreted probabilistically. No assessment of potential interactions with other domains is planned.

**TABLE 4:** Analyses of heterogeneity of intervention effects for the primary outcome.

Baseline characteristics	Definition and type	Expected direction of interaction
Severity of illness	Severity of illness according to the Simplified Mortality Score for the Intensive Care Unit [28] (SMS‐ICU); continuous.	Larger beneficial effect of albumin in patients with more severe illness.
Primary cause of shock	Septic, cardiogenic, haemorrhagic or traumatic versus other types of shock (e.g., neurogenic, anaphylactic, burn and obstructive shock); categorical.	Larger beneficial effect of albumin in patients with septic shock compared with other types of shock.
Albumin levels	Plasma albumin at baseline; continuous.	Larger beneficial effect of albumin in patients with lower baseline plasma albumin.
Renal function	Highest plasma creatinine value in the 24 h before randomisation; continuous.	Larger beneficial effect of albumin in patients with worse baseline renal function (higher plasma creatinine).
Liver failure	Patients with chronic liver failure versus patients with normal liver function; categorical.	Larger beneficial effect of albumin in patients with chronic liver failure.
Baseline IV fluids	IV crystalloid volumes administered in the 24 h before randomisation; continuous.	Larger beneficial effect of albumin in patients who received more IV fluid before randomisation.

*Note:* Heterogeneity of intervention effects will be assessed for the primary outcome according to the baseline characteristics outlined in this table. For continuous baseline variables (severity of illness, albumin levels, renal function, baseline IV fluids), heterogenous intervention effects will be assessed using linear regression models similar to the primary model, but also including the baseline characteristic of interest, using linear and quadratic terms and their interactions with the intervention group (after mean‐centring and, for variables expected to be substantially right‐skewed, log2‐transformation). Mean values for the primary outcome will be predicted in each intervention group across the full range of values of the baseline variable of interest using a G‐computation approach described in the full domain‐specific appendix [18] and used to calculate differences on the absolute and relative scales. For categorical baseline variables (primary cause of shock and liver failure), hierarchical linear regression models similar to the primary models but with random effects for the intercept and intervention effect terms in each level of the category. These models will be used to make predictions for each intervention arm in each level of the category, similar to the models used for continuous baseline variables.

Abbreviations: IV: intravenous; SMS‐ICU: Simplified Mortality Score for the Intensive Care Unit [28].

#### 2.7.6 Missing Data Handling

Proportions of missing data will be presented, and missingness handled using multiple imputation [33] with best‐worst/worst‐best case sensitivity analyses; details are outlined in the core protocol and the full domain‐specific appendix [17, 18].

#### 2.7.7 Statistical Simulation and Performance Metrics

The *INCEPT‐Albumin* domain design has been developed and evaluated using statistical simulation via R v4.4.1 (R Core Team, R Foundation for Statistical Computing, Vienna, Austria) with the *adaptr* [34] R package (https://inceptdk.github.io/adaptr) following the approach previously described and used [24, 26, 35]. The final design as well as sensitivity analyses with different design choices and key assumptions were all evaluated using 100,000 simulations under multiple clinical scenarios. In brief, we assumed that the distribution of the primary and guiding outcome in the no albumin use arm resembled the distribution from the *CLASSIC* trial [3]. This reference distribution was modelled using a *binomial* distribution handling the probability of having 0 or > 0 days, and a *beta* distribution modelling the distribution amongst those with > 0 days. In summary, 40.2% had 0 days (36.7% due to death), with an estimated mean of 23.5 days in those with > 0 days, and an overall estimated mean of 14 days. Simulations were conducted in scenarios with no difference between arms, and with small and large differences in both directions in the albumin arm, corresponding to overall mean differences of 1 or 5 days in either direction. Multiple performance metrics [24, 26] were evaluated, with key metrics presented in Table 5. The expected sample sizes across scenarios ranged from 1137 to 3547 participants, with approximately 100% probabilities of conclusiveness across scenarios.

**TABLE 5:** Key performance metrics of the final domain design.

Metric	No difference	Small benefit with albumin	Small harm with albumin	Large benefit with albumin	Large harm with albumin
Sample size—mean [25; 50; 75%‐percentiles]	2971 [2136; 2637; 3387]	3437 [2136; 3135; 4635]	3247 [2136; 2886; 4386]	1137 [1137; 1137; 1137]	1137 [1137; 1137; 1137]
Mean number of days alive without life support at 30 days—mean [25; 50; 75%‐percentiles]	14.5 days [14.3; 14.5; 14.6]	15.0 days [14.9; 15.0; 15.2]	14 days [13.9; 14.0; 14.2]	16.9 days [16.6; 16.9; 17.2]	12.0 days [11.7; 12.0; 12.2]
Probability of conclusiveness	~100.0%	~100.0%	~100.0%	~100.0%	~100.0%
Probability of superiority	5.0%	71.8%	75.5%	~100.0%	~100.0%
Probability of equivalence	95.0%	28.2%	24.5%	~0.0%	~0.0%
Probability of inconclusiveness	~0.0%	~0.0%	~0.0%	~0.0%	~0.0%
Root mean squared error (for estimated mean number of days in the superior arm, for simulations stopped for superiority only)	0.8 days	0.4 days	0.4 days	0.6 days	0.5 days
Probability of stopping for superiority for the *no albumin* arm	2.7%	~0.0%	75.4%	~0.0%	~100.0%
Probability of stopping for superiority for the *albumin* arm	2.3%	71.8%	~0.0%	~100.0%	~0.0%

*Note:* Key performance metrics of the final *INCEPT‐Albumin* domain design under the primary assumptions used based on 100,000 simulations for each scenario. The probabilities of conclusiveness correspond to the combined probabilities of superiority and practical equivalence; the probabilities of superiority may be interpreted as the type 1 error rate in the scenario with no difference and as the power in the scenarios with differences present [24]. The probabilities of inconclusiveness refers to the proportion of simulated trials stopped at the maximum sample size without triggering any stopping rule.

### 2.8 Stakeholder Involvement

Stakeholders were involved according to the requirements outlined in the core protocol [17, 18]. Two ICU survivors, one family member, and three clinicians not involved in planning or daily management of the domain were involved in discussions of domain outcomes including the primary/guiding outcome. Three ICU survivors, one family member, and 10 clinicians not involved in planning or daily management of *INCEPT* provided inputs to the lay summary describing the domain and all written informed consent materials. Stakeholders were involved through an established research panel [36] via group discussions according to the nominal group technique [37]. Future stakeholder involvement is planned for the dissemination phase.

### 2.9 Organisational Aspects, Independent Data Monitoring and Safety Committee, and Monitoring

Daily management of *INCEPT‐Albumin* is the responsibility of a domain sponsor and management committee, referring to the *INCEPT* platform sponsor and management committee. The domain is externally monitored according to the principles outlined in the core protocol [17], and domain conduct and safety are overseen by a multidisciplinary independent data monitoring and safety committee according to the principles outlined in the core protocol and full domain‐specific appendix [17].

### 2.10 Dissemination

We plan to present results from the integrated feasibility phase, for short‐term outcomes (30 days), and longer‐term (90–180 days) outcomes separately as preprints (for clinical outcomes), on the trial website, and in international peer‐reviewed medical journals, according to the *CONSORT‐ACE* guideline [20].

## 3 Discussion

The *INCEPT‐Albumin* domain compares the effects of albumin versus no albumin for resuscitation and substitution in adult acutely admitted ICU patients with shock on days alive without life support at 30 days and other patient‐important outcomes. Due to the pragmatic design, large maximum sample size, and the adaptive design, *INCEPT‐Albumin* will with high probability provide results directly informing clinical practice, without continuing longer than necessary.

### 3.1 Strengths

*INCEPT‐Albumin* was clinically informed by data from a previous fluid trial [15] and a large international survey [6]. The strengths of *INCEPT* in general have been discussed elsewhere [17]; they also apply to *INCEPT‐Albumin*. In brief, they include high generalisability and external validity due to participation of ICUs across many countries; the pragmatic design, ensuring clinical relevance; the large maximum sample size and adaptations, ensuring a high probability of obtaining conclusive evidence without continuing longer than necessary; the use of (restricted) response‐adaptive randomising, ensuring that the domain ‘learns’ from accruing data, increasing probabilities of better outcomes for participants, while mitigating potential downsides of response‐adaptive randomisation [16, 24, 26]; the prospectively planned stakeholder involvement; the use of Bayesian statistical methods with probabilistic interpretation of results; and the thorough assessment of design choices and assumptions using statistical simulation [17].

### 3.2 Limitations

*INCEPT‐Albumin* also has limitations. First, the open‐label design may introduce a risk of bias. As the primary and most of the secondary outcomes are *hard* endpoints (except health‐related quality of life and cognitive function), the risk of bias is considered small; while knowledge of the allocated intervention may affect clinical decision making, including initiation, continuation, or discontinuation of life support, these decisions typically involve multiple ICU clinicians and are influenced by objective measures. Similarly, knowledge of the intervention may affect clinicians' use of co‐interventions. However, blinding this domain would be costly, time‐consuming, and logistically challenging due to the different albumin solutions and the many sites. As the risk of bias is considered small, we consequently have chosen an open‐label design capable of recruiting a larger sample and thus more likely to provide adequately precise results with higher probabilities of conclusiveness. For similar reasons, we are not registering co‐interventions or reasons for initiating or withdrawing life support. Second, the choice of primary outcome could be challenged, given the focus in previous trials on albumin on mortality [8]. We, however, consider it likely that albumin may affect the requirements for life support without necessarily affecting mortality enough for this to be detectable in a moderate to large sized trial. The use of *days alive without life support* is in line with recent increases in the use of this outcome in other trials conducted in the critically ill [38], and recent recommendations to focus on more granular outcomes, including days alive without life support, as these outcomes are both patient‐important [36] and provide more statistical information than mortality, increasing the probabilities of conclusive results [39]. Third, the use of response‐adaptive randomisation in an open‐label trial can be discussed [40]; to avoid prematurely affecting equipoise, allocation probabilities are restricted to minimum 40% and exact allocation probabilities are not revealed during domain conduct. Forth, as the intervention protocols are pragmatic, there is a risk that adequate separation will not be obtained. Fifth, as the intervention period spans a maximum of 90 days, the risk of protocol violations may be substantial. The latter two limitations are assessed in the integrated feasibility phase, providing an opportunity to modify the protocol or update trial procedures if relevant. Finally, to minimise the data collection burden, we will not register indications for each albumin administration.

## 4 Conclusions

*INCEPT‐Albumin* domain will with high probability provide conclusive evidence on the comparative effectiveness of albumin versus no albumin for resuscitation and substitution in adult acutely admitted ICU patients with shock, measured by days alive without life support at 30 days and other patient‐important outcomes. This is expected to directly inform clinical practice.

## Author Contributions

Conceptualisation: T.S.M., A.P., P.S., K.L.E., M.H.M., A.G. Funding acquisition: T.S.M., A.P., T.L., F.K., M.H.M., A.G. Investigation: all authors. Methodology: all authors. Project administration: T.S.M., A.P., R.F.L., M.‐B.N.K., B.S.K.‐H., M.H.M., A.G. Software: A.K.G.J., B.S.K.‐H., T.L., A.G. Supervision: A.P., M.H.M., A.G. Writing – original draft: T.S.M., A.P., P.S., K.L.E., A.G; writing – review and editing: all authors.

## Funding

*INCEPT* is funded by the *Novo Nordisk Foundation* (NNF23OC0085106) and *Sygeforsikringen ‘danmark’* (2020‐0320), with additional support from *Grosserer Jakob Ehrenreich og Hustru Grete Ehrenreichs Fond*, *Dagmar Marshalls Fond*, and *Savværksejer Jeppe Juhl og hustru Ovita Juhls Mindelegat*. The *INCEPT‐Albumin* domain is partially funded by *Danmarks Frie Forskningsfond* (4308‐00210B). None of the funders have had any influence on the planning of the platform trial, and none will have any influence on the design, conduct, analysis, or reporting of any domains assessed on the platform. None of the funders will have ownership of any trial data.

## Conflicts of Interest

Tine Sylvest Meyhoff: *coordinating investigator of the CLASSIC trial (NCT03668236) which was supported by a grant (NNF17OC0028608) from the Novo Nordisk Foundation and by the Sofus Friis' Foundation, Rigshospitalet's Research Council, and the Danish Society of Anesthesiology and Intensive Care Medicine*. Anders Perner: *research grants from Novo Nordisk Foundation, Sygeforsikringen ‘danmark’*, *and Savværksejer Jeppe Juhl and Danmarks Frie Forskningsfond. Honorarium for advisory board work from Novartis*. Praleene Sivapalan: *coordinating investigator of the CLASSIC trial (NCT03668236) which was supported by a grant (NNF17OC0028608) from the Novo Nordisk Foundation and by the Sofus Friis' Foundation, Rigshospitalet's Research Council, and the Danish Society of Anesthesiology and Intensive Care Medicine. Grantsfrom Rigshospitalet's Research Council in 2020 and Grosserer Jakob Ehrenreich og Hustru Grete Ehrenreichs Fond in 2022*. Maj‐Brit Nørregaard Kjær: *funding from the Research Council of Rigshospitalet*. Benjamin Skov Kaas‐Hansen: *grant from Grosserer Jakob Ehrenreich og Hustru Grete Ehrenreichs Fond in 2022 and a Danish Data Science Academy postdoc fellowship from mid‐December 2024*. Theis Lange: *served on the data monitoring committee for industry studies (Novo Nordisk and Leo Pharma), therapeutic areas not related to intensive care*. Carmen Andrea Pfortmueller: *no personal conflicts of interest (personal financial interest). The department of Intensive Care at Inselspital, University Hospital of Bern report grants from Orion Pharma, Abbott Nutrition International, B. Braun Medical AG, CSEM AG, Edwards Lifesciences Services GmbH, Kenta Biotech Ltd, Maquet Critical Care AB, Omnicare Clinical Research AG, Nestle, Pierre Fabre Pharma AG, Pfizer, Bard Medica S.A., Abbott AG, Anandic Medical Systems, Pan Gas AG Healthcare, Bracco, Hamilton Medical AG, Fresenius Kabi, Getinge Group Maquet AG, Dräger AG, Teleflex Medical GmbH, Glaxo Smith Kline, Merck Sharp and Dohme AG, Eli Lilly and Company, Baxter, Astellas, Astra Zeneca, CSL Behring, Novartis, Covidien, and Nycomed outside the submitted work. The money was paid into departmental funds; no personal financial gain applied*. All other authors: no relevant conflicts of interest.

## Data Availability

No patient data were analysed in this protocol manuscript. Data sharing plans for the domain are described in the domain‐specific appendix and core protocol available at the trial website (www.incept.dk).

## References

[1] J.‐L. Vincent and D. De Backer , “Circulatory Shock,” New England Journal of Medicine 369 (2013): 1726–1734, 10.1056/NEJMra1208943. 24171518

[2] Y. Sakr , K. Reinhart , J. L. Vincent , et al., “Does Dopamine Administration in Shock Influence Outcome? Results of the Sepsis Occurrence in Acutely Ill Patients (SOAP) Study,” Critical Care Medicine 34 (2006): 589–597, 10.1097/01.CCM.0000201896.45809.E3. 16505643

[3] T. S. Meyhoff , P. B. Hjortrup , J. Wetterslev , et al., “Restriction of Intravenous Fluid in ICU Patients With Septic Shock,” New England Journal of Medicine 386 (2022): 2459–2470, 10.1056/NEJMoa2202707. 35709019

[4] M.‐B. N. Kjær , T. S. Meyhoff , P. Sivapalan , et al., “Long‐Term Effects of Restriction of Intravenous Fluid in Adult ICU Patients With Septic Shock,” Intensive Care Medicine 49 (2023): 820–830, 10.1007/s00134-023-07114-8. 37330928 PMC10354110

[5] J. A. Myburgh and M. G. Mythen , “Resuscitation Fluids,” New England Journal of Medicine 369 (2013): 1243–1251, 10.1056/NEJMra1208627. 24066745

[6] P. Sivapalan , K. L. Ellekjaer , A. Perner , et al., “Preferences for Albumin Use in Adult Intensive Care Unit Patients With Shock: An International Survey,” Acta Anaesthesiologica Scandinavica 68 (2024): 1234–1243, 10.1111/aas.14479. 39302760

[7] J. L. Vincent , M. J. Dubois , R. J. Navickis , and M. M. Wilkes , “Hypoalbuminemia in Acute Illness: Is There a Rationale for Intervention? A Meta‐Analysis of Cohort Studies and Controlled Trials,” Annals of Surgery 237 (2003): 319–334, 10.1097/00000658-200303000-00005. 12616115 PMC1514323

[8] S. R. Lewis , M. W. Pritchard , D. J. Evans , et al., “Colloids Versus Crystalloids for Fluid Resuscitation in Critically Ill Patients,” Cochrane Database of Systematic Reviews 8 (2018): CD000567, 10.1002/14651858.CD000567.pub7. 30073665 PMC6513027

[9] G. S. Martin and P. Bassett , “Crystalloids vs. Colloids for Fluid Resuscitation in the Intensive Care Unit: A Systematic Review and Meta‐Analysis,” Journal of Critical Care 50 (2019): 144–154, 10.1016/j.jcrc.2018.11.031. 30540968

[10] P. Caironi , G. Tognoni , S. Masson , et al., “Albumin Replacement in Patients With Severe Sepsis or Septic Shock,” New England Journal of Medicine 370 (2014): 1412–1421, 10.1056/nejmoa1305727. 24635772

[11] The SAFE Study Investigators , “A Comparison of Albumin and Saline for Fluid Resuscitation in the Intensive Care Unit,” New England Journal of Medicine 350 (2004): 2247–2256, 10.1056/NEJMoa040232. 15163774

[12] L. Evans , A. Rhodes , W. Alhazzani , et al., “Surviving Sepsis Campaign: International Guidelines for Management of Sepsis and Septic Shock 2021,” Intensive Care Medicine 47 (2021): 1181–1247, 10.1007/s00134-021-06506-y. 34599691 PMC8486643

[13] J. Callum , N. J. Skubas , A. Bathla , et al., “Use of Intravenous Albumin: A Guideline From the International Collaboration for Transfusion Medicine Guidelines,” Chest 166 (2024): 321–338, 10.1016/j.chest.2024.02.049. 38447639 PMC11317816

[14] Y. M. Arabi , E. Belley‐Cote , A. Carsetti , et al., “European Society of Intensive Care Medicine Clinical Practice Guideline on Fluid Therapy in Adult Critically Ill Patients. Part 1: The Choice of Resuscitation Fluids,” Intensive Care Medicine 50 (2024): 813–831, 10.1007/s00134-024-07369-9. 38771364

[15] T. S. Meyhoff , A. Granholm , P. B. Hjortrup , et al., “Albumin Use in Patients With Septic Shock—Post‐Hoc Analyses of an International Randomised Fluid Trial,” Acta Anaesthesiologica Scandinavica 68 (2023): 372–384, 10.1111/aas.14359. 37975538

[16] Adaptive Platform Trials Coalition , “Adaptive Platform Trials: Definition, Design, Conduct and Reporting Considerations,” Nature Reviews. Drug Discovery 18 (2019): 797–807, 10.1038/s41573-019-0034-3. 31462747

[17] A. Granholm , M. H. Møller , B. S. Kaas‐Hansen , et al., “INCEPT: The Intensive Care Platform Trial—Design and Protocol,” Acta Anaesthesiologica Scandinavica 69 (2025): e70023, 10.1111/aas.70023. 40084471 PMC11907384

[18] INCEPT , “The Intensive Care Platform Trial,” 2025, accessed December 1, 2025, https://www.incept.dk.

[19] A.‐W. Chan , I. Boutron , S. Hopewell , et al., “SPIRIT 2025 Statement: Updated Guideline for Protocols of Randomised Trials,” BMJ 389 (2025): e081477, 10.1136/bmj-2024-081477. 40294953 PMC12035670

[20] M. Dimairo , P. Pallmann , J. Wason , et al., “The Adaptive Designs CONSORT Extension (ACE) Statement: A Checklist With Explanation and Elaboration Guideline for Reporting Randomised Trials That Use an Adaptive Design,” BMJ 369 (2020): m115, 10.1136/bmj.m115. 32554564 PMC7298567

[21] A. Granholm , B. S. Kaas‐Hansen , T. Lange , et al., “Use of Days Alive Without Life Support and Similar Count Outcomes in Randomised Clinical Trials – An Overview and Comparison of Methodological Choices and Analysis Methods,” BMC Medical Research Methodology 23 (2023): 139, 10.1186/s12874-023-01963-z. 37316785 PMC10266319

[22] M. Herdman , C. Gudex , A. Lloyd , et al., “Development and Preliminary Testing of the New Five‐Level Version of EQ‐5D (EQ‐5D‐5L),” Quality of Life Research 20 (2011): 1727–1736, 10.1007/s11136-011-9903-x. 21479777 PMC3220807

[23] Z. S. Nasreddine , “MoCA Test: Validation of a Five‐Minute Telephone Version,” Alzheimer's & Dementia 17 (2021): e057817, 10.1002/alz.057817.

[24] A. Granholm , B. S. Kaas‐Hansen , T. Lange , et al., “An Overview of Methodological Considerations Regarding Adaptive Stopping, Arm Dropping, and Randomization in Clinical Trials,” Journal of Clinical Epidemiology 153 (2023): 45–54, 10.1016/j.jclinepi.2022.11.002. 36400262

[25] E. C. Goligher , A. Heath , and M. O. Harhay , “Bayesian Statistics for Clinical Research,” Lancet 404 (2024): 1067–1076, 10.1016/S0140-6736(24)01295-9. 39277290 PMC12051211

[26] A. Granholm , A. K. G. Jensen , T. Lange , A. Perner , M. H. Møller , and B. S. Kaas‐Hansen , “Designing and Evaluating Bayesian Advanced Adaptive Randomised Clinical Trials: A Practical Guide,” Pharmaceutical Statistics 24 (2025): e70042, 10.1002/pst.70042. 41065317 PMC12509790

[27] United States Food and Drug Administration , “Adaptive Designs for Clinical Trials of Drugs and Biologics ‐ Guidance for Industry,” Guidance Document, 2019, accessed February 27, 2025, https://www.fda.gov/regulatory‐information/search‐fda‐guidance‐documents/adaptive‐design‐clinical‐trials‐drugs‐and‐biologics‐guidance‐industry.

[28] A. Granholm , A. Perner , M. Krag , et al., “Development and Internal Validation of the Simplified Mortality Score for the Intensive Care Unit (SMS‐ICU),” Acta Anaesthesiologica Scandinavica 62 (2018): 336–346, 10.1111/aas.13048. 29210058

[29] K. Rockwood , “A Global Clinical Measure of Fitness and Frailty in Elderly People,” Canadian Medical Association Journal 173 (2005): 489–495, 10.1503/cmaj.050051. 16129869 PMC1188185

[30] K. Rockwood and O. Theou , “Using the Clinical Frailty Scale in Allocating Scarce Health Care Resources,” Canadian Geriatrics Journal 23 (2020): 210–215, 10.5770/cgj.23.463. 32904824 PMC7458601

[31] United States Food and Drug Administration , “Adjusting for Covariates in Randomized Clinical Trials for Drugs and Biological Products,” Guidance Document, 2023, https://www.fda.gov/regulatory‐information/search‐fda‐guidance‐documents/adjusting‐covariates‐randomized‐clinical‐trials‐drugs‐and‐biological‐products.

[32] T. J. Iwashyna , J. F. Burke , J. B. Sussman , H. C. Prescott , R. A. Hayward , and D. C. Angus , “Implications of Heterogeneity of Treatment Effect for Reporting and Analysis of Randomized Trials in Critical Care,” American Journal of Respiratory and Critical Care Medicine 192 (2015): 1045–1051, 10.1164/rccm.201411-2125CP. 26177009 PMC4642199

[33] S. van Buuren , Flexible Imputation of Missing Data, 2nd ed. (CRC Press, 2018), https://stefvanbuuren.name/fimd.

[34] A. Granholm , A. K. G. Jensen , T. Lange , and B. S. Kaas‐Hansen , “Adaptr: An R Package for Simulating and Comparing Adaptive Clinical Trials,” Journal of Open Source Software 7 (2022): 4284, 10.21105/joss.04284.

[35] A. Granholm , M. W. Munch , N. Meier , et al., “Empirical Meropenem Versus Piperacillin/Tazobactam for Adult Patients With Sepsis (EMPRESS) Trial: Protocol,” Acta Anaesthesiologica Scandinavica 68 (2024): 1107–1119, 10.1111/aas.14441. 38769040

[36] M.‐B. N. Kjær , C. R. L. Bruun , A. Granholm , et al., “A Core Outcome Set for Adult General ICU Patients,” Critical Care Medicine 53 (2025): e575–e589, 10.1097/CCM.0000000000006556. 40036020

[37] S. S. McMillan , M. King , and M. P. Tully , “How to Use the Nominal Group and Delphi Techniques,” International Journal of Clinical Pharmacy 38 (2016): 655–662, 10.1007/s11096-016-0257-x. 26846316 PMC4909789

[38] A. Granholm , C. T. Anthon , M.‐B. N. Kjær , et al., “Patient‐Important Outcomes Other Than Mortality in Contemporary ICU Trials: A Scoping Review,” Critical Care Medicine 50 (2022): e759–e771, 10.1097/CCM.0000000000005637. 35894598

[39] M. O. Harhay , J. Wagner , S. J. Ratcliffe , et al., “Outcomes and Statistical Power in Adult Critical Care Randomized Trials,” American Journal of Respiratory and Critical Care Medicine 189 (2014): 1469–1478, 10.1164/rccm.201401-0056CP. 24786714 PMC4226016

[40] D. S. Robertson , K. M. Lee , B. C. López‐Kolkovska , and S. S. Villar , “Response‐Adaptive Randomization in Clinical Trials: From Myths to Practical Considerations,” Statistical Science 38 (2023): 185–208, 10.1214/22-STS865. 37324576 PMC7614644

